# Hardy-Weinberg equilibrium revisited for inferences on genotypes featuring allele and copy-number variations

**DOI:** 10.1038/srep09066

**Published:** 2015-03-13

**Authors:** Andreas Recke, Klaus-Günther Recke, Saleh Ibrahim, Steffen Möller, Reinhard Vonthein

**Affiliations:** 1 Lübeck Institute of Experimental Dermatology, University of Lübeck, Lübeck, Germany; 2 Department of Dermatology, Allergology and Venereology, University of Lübeck, Lübeck, Germany; 3 Institute of Medical Biometry and Statistics, University of Lübeck, Lübeck, Germany; 4 Center for Clinical Studies, University of Lübeck, Lübeck, Germany

## Abstract

Copy number variations represent a substantial source of genetic variation and are associated with a plethora of physiological and pathophysiological conditions. Joint copy number and allelic variations (CNAVs) are difficult to analyze and require new strategies to unravel the properties of genotype distributions. We developed a Bayesian hidden Markov model (HMM) approach that allows dissecting intrinsic properties and metastructures of the distribution of CNAVs within populations, in particular haplotype phases of genes with varying copy numbers. As a key feature, this approach incorporates an extension of the Hardy-Weinberg equilibrium, allowing both a comprehensive and parsimonious model design. We demonstrate the quality of performance and applicability of the HMM approach with a real data set describing the Fc*γ* receptor (Fc*γ*R) gene region. Our concept, using a dynamic process to analyze a static distribution, establishes the basis for a novel understanding of complex genomic data sets.

Copy number variations (CNVs) are common and represent a source of enormous genetic complexity, which is further increased by sequence variations. CNVs have been recognized as highly important for the understanding of human disease pathogenesis^1^. Nonetheless, the complexity of CNAV poses a challenge for statistical association analyses, requiring novel approaches to reveal hidden metastructures.

Most CNVs are inherited, but 10% develop *de novo*, either during parental meiosis (30%) or during developmental mitosis (70%). The majority of CNVs (approximately 80%) are gains^2^. During mitosis, several mechanisms have been proposed to cause changes in copy numbers^3^,^4^,^5^,^6^. Among these mechanisms, the microhomology-mediated break-induced repair (MMBIR) mechanism has been proposed to be a major source of CNVs^4^,^7^. This repair mechanism uses either the sister DNA strand or the second chromosome as a reference to repair DNA strand breaks during replication. In microhomologous regions, annealing to the reference may be misplaced, leading to either the deletion or duplication of the affected gene region. This mechanism causes loss of heterozygosity (LOH) as its specific signature in the respective DNA sequence^4^,^7^.

A prominent example of a CNAV is in the genetic region harboring the genes for low-affinity Fc*γ* receptors. Fc*γ* receptors are key molecules for the binding of immunoglobulins by cellular players of the immune system and mediate a plethora of downstream signaling events^8^,^9^. These receptors are associated with susceptibility to autoimmune diseases, including systemic lupus erythematosus, rheumatoid arthritis and idiopathic thrombocytopenic purpura^10^,^11^,^12^,^13^,^14^,^15^,^16^. Although each Fc*γ*R possesses a unique functionality, their respective genes show a very high intergenic homology. Currently, the most advanced high-throughput method to characterize CNAVs of the Fc*γ*R gene region is a multiplex ligation-dependent probe amplification (MLPA) method^16^,^17^. This method determines the abundance counts of sequence motifs, i.e., alleles, in genomic DNA. In detail, this method determines integer copy numbers for 7 genes within the Fc*γ*R gene region, ranging from 0 to more than 5 copies, thereby distinguishing allelic variants of 9 different single nucleotide polymorphisms (SNPs) (Fig. 1).

The approach we present here was originally developed to address the complexity of this data set. For this purpose, we re-interpret these data as the static summary of a dynamic series of events. The order of events is introduced as a latent variable described by a specialized hidden Markov model (HMM) that is randomly walked according to transition probabilities, which are inferred from the data set. The order of events can be regarded as the order of genes and alleles along a single chromosomal strand (Fig. 2a). Inspired by the above described mechanisms that lead to copy number variation, we introduce recursive loops that model deletion or multiplication of genes (Fig. 2b).

The Hardy-Weinberg equilibrium is a widely used model to describe the distribution of genotypes without copy-number variation in a population. It is based upon the idealized assumption that genetic alleles are independent and distribute evenly with every generation in a population. We extend this idea by further assuming independence and even distribution of alleles between gene copies. Moreover, we assume that the processes that lead to copy-number variation have reached a steady-state equilibrium. This leads to the Markov model graph described in Fig. 2c. The above described CNAV generating mechanisms that cause the LOH signature can be incorporated by further derivation of this model (Fig. 2d).

The Markov model graph (Fig. 2b–d) and the genotyping data are given as inputs to a Bayesian frame-work that infers the unknown transition probabilities and latent Markov path. For the selection of the best HMM, the marginal likelihood is also calculated.

Here, we present a novel approach for statistical inferences of complex genotypes within a population together with a collection of applications of the method to a real MLPA data set. We demonstrate how an optimal HMM input graph can be set up for different purposes and how the results of Bayesian inference can be analyzed.

## Results

To derive transition probabilities from given observed data and a Markov model graph, a Markov Chain Monte Carlo (MCMC) sampler, i.e. a data-augmented Metropolis-within-Gibbs sampler, alternately simulates latent Markov paths and transition probabilities. The implementation details are further described in the Methods and Suppl. Materials sections. The output of the sampler can be used to determine credibility intervals and point estimators for transition probabilities for the respective input HMM graph, as shown below.

### HMM interpretation of the Hardy-Weinberg equilibrium model

To introduce our approach, we first apply it to two Fc*γ*R genes with two allelic variants that do not show any copy number variation. The models depicted in Fig. 3a–b were designed to reflect the genotype distribution of Fc*γ*R IIa (Fig. 3a) and Fc*γ*R IIb (Fig. 3b). This basic model performs the same function as the calculations for a regular Hardy-Weinberg equilibrium (Suppl. Tab. 1). The transition probabilities at the bifurcation point in the models are therefore identical to the haplotype frequencies for the H131R polymorphism (rs1801274) of Fc*γ*R IIa and the I232T polymorphism (rs1050501) of Fc*γ*R IIb.

### Extension of the Hardy-Weinberg equilibrium model to joint copy-number and allelic variations

**Figure 1:**
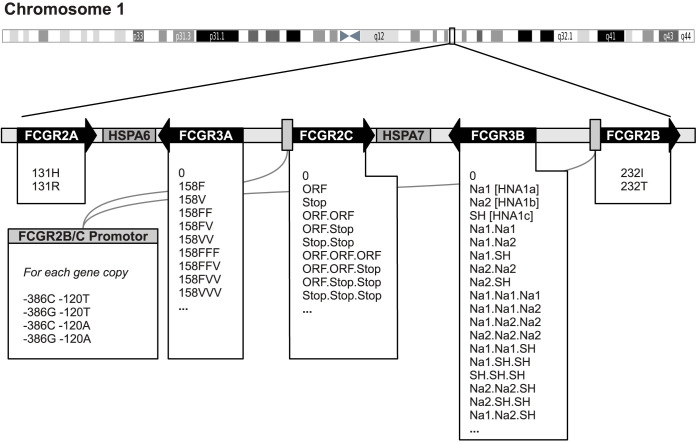
Schematic of the genetic information measured by the Fc*γ* receptor multiplex ligation-dependent amplification (MLPA) system. The Fc*γ*R-specific MLPA system determines copy numbers of depicted gene alleles of Fc*γ* receptors IIa (FCGR2A, rs1801274), IIb (FCGR2B, rs1050501), IIc (FCGR2C, rs183547105), IIIa (FCGR3A, rs396911) and IIIb (FCGR3B, rs200688856 and rs5030738). Additionally, copy numbers of heat shock proteins A6 (HSPA6) and A7 (HSPA7) are determined. For Fc*γ*R IIb and IIc, the promoter sequences are highly homologous. Thus, allele counts of the two promoter polymorphisms C-386G (rs3219018) and A-120T (rs34701572) are not separable between these receptors.

The genes for Fc*γ*R IIIa, IIc and IIIb show both allelic and copy number variations (compare Fig. 1). To establish models that describe the CNAVs in these receptors, we focus on those with the F158V amino acid polymorphism of Fc*γ*R IIIa (Fig. 3c); the *open reading frame* (ORF) *stop codon* polymorphisms of Fc*γ* receptor IIc (Fig. 3d); and the HNA1a (Na1), HNA1b (Na2) and HNA1c (SH) polymorphisms of Fc*γ* receptor IIIb (Fig. 3e).

**Figure 2:**
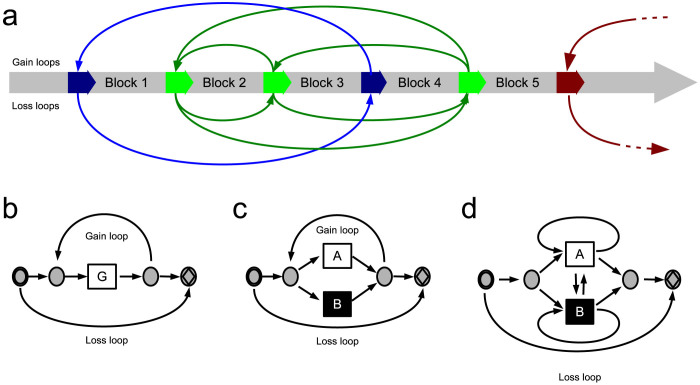
Derivation of a stochastic finite-state machine that simulates copy-number and allelic variation (CNAV). (a), general concept to describe variable repetitions or deletions in chromosomal DNA: the chromosomal DNA strand (gray blocks) contains different microhomologous sites (thick arrows in blue, green and red) that act as anchors for DNA strand repairing mechanisms and crossing-over during meiosis. Mismatches of these sites lead to the losses or duplications (bended arrows) of gene blocks, i.e., copy-number variation. (b), definition of a stochastic finite-state machine that reproduces the situation of a single block harboring gene **G** in a single chromosome or haplotype. Beginning at the initial state (circle-within-circle state), the machine walks the graph randomly (transition probabilities are not shown here), either emitting a symbol (rectangle states) or nothing (circle states) until it reaches the final state (circle-diamond states). Every genotype consists of two haplotypes; thus, the machine walks the graph twice to produce a complete genotype. Loss and gain loops are separated here to avoid infinite recursion. (c), further derivation of the model for representation of a gene with two alleles (A and B), assuming independence between changes in copy numbers and between individual gene copies. (d), a model for a biallelic gene that incorporates a possible loss of heterozygosity (LOH).

To set up a model for these genes, we extend the basic assumption of the Hardy-Weinberg equilibrium, i.e., independence of alleles between chromosomes, by assuming 2 other independences: independence between individual gene copies and independence between copy number changing events (compare Fig. 2c). This approach leads to an HMM as outlined in Fig. 2b and c. The changes in copy numbers are represented by two separate loops for gains and losses of gene copy numbers. To obtain an impression of the predictive capability of this model, the marginal likelihood is calculated according to the Chib and Jeliazkov method (CJ method)^18^ and compared to that of a naïve enumeration model (NEM, see the Methods section) by the Bayes factor.

**Figure 3:**
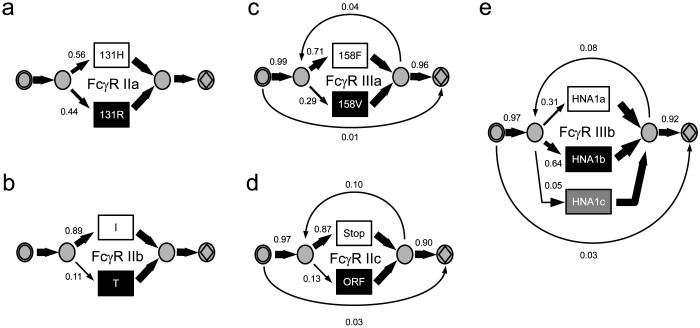
HMM representation of copy number and allelic variation in Fc*γ*R genes. (a), directed HMM graph for the H131R amino acid polymorphism of Fc*γ*R IIa, (b), directed HMM graph for the I232T amino acid polymorphism of Fc*γ*R IIb. Both Fc*γ*R IIa and IIb do not exhibit copy number variation. (c), HMM for Fc*γ*R IIIa, including loops for null alleles and multiplication, as well as the F158V amino acid polymorphism. (d), HMM for Fc*γ*R IIc with the open reading frame (ORF) and stop codon polymorphism. (e), HMM for Fc*γ* receptors IIIb with the HNA1a (Na1), HNA1b (Na2) and HNA1c (SH) allelic variation. Transition probabilities are indicated next to the transition edges and further by edge thickness and color. Circles (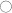) indicate silent states, and boxes (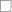) indicate states that emit a counting event as denoted inside. The starting state is indicated by a circle-within-circle, the final state by a diamond-within-circle.

In the case of Fc*γ*R IIIa, a Bayes factor (HMM over NEM) of 66.7 favors the HMM (Fig. 3c). The same is true for Fc*γ*R IIc, with an even higher Bayes factor of 8103 (HMM over NEM)(Fig. 3d). The sampler output of 10 separate runs for Fc*γ*R IIIa and Fc*γ*R IIc shows rapid convergence to a single optimum, with almost no autocorrelation (Suppl. Figs. 1 and 2).

Both examples support the extended Hardy-Weinberg equilibrium model. Moreover, the HMM predicts the frequency of all theoretically possible genotypes by design, even those not found in the observed data set. Enumeration models do not have this predictive capability, which is known as the *zero-frequency* problem^19^. Therefore, comparison between HMMs and NEM is, strictly speaking, inadequate. We nevertheless present this comparison here to provide a rough orientation to the performance of the respective HMMs.

As described, changes in copy numbers can be caused by the MMBIR mechanism, which would violate the independence assumption between individual gene copies, as LOH would be introduced. We thus compare the performance of HMMs incorporating LOH (compare Fig. 2d) for both Fc*γ*R IIIa and IIc (Suppl. Fig. 3a and b). However, according to a comparison by the Bayes factor, the LOH models both performed slightly worse than the corresponding models without LOH, indicating no advantage for the extra complexity introduced in the LOH models.

In contrast to Fc*γ* receptor IIIa and IIc, the basic HMM for Fc*γ*R IIIb (Fig. 3e) appears to be worse than the NEM, with a Bayes factor of 2.3 × 10^−27^. This finding indicates that the genotype distribution of this receptor violates some of the equilibrium assumptions described above.

All calculations were done on a desktop computer with an Intel Core i5 quad-core CPU 660 @ 3.33 GHz with 8 GB RAM. For our data set with 387 individuals, the average running time for 1000 sampling cycles of the MCMC sampler was about 65 seconds for each of the models described in Fig. 3a–b and about 80 seconds for those described in Fig. 3c–e.

### Using the HMM approach to model genotype distribution disequilibria

**Figure 4:**
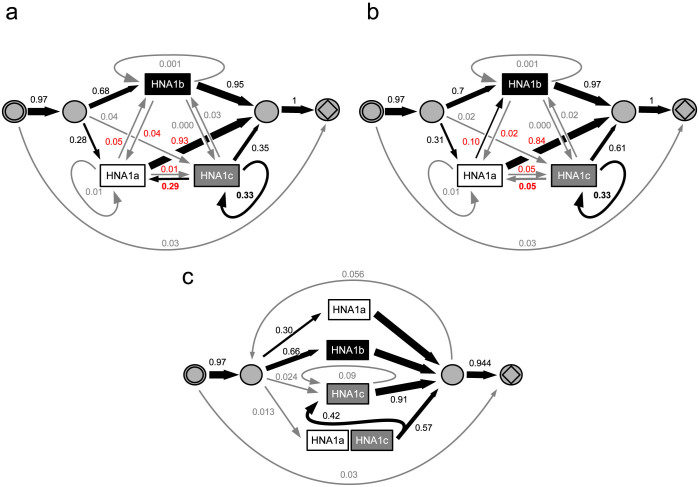
Improving the HMM for Fc*γ*R IIIb by incorporating non-equilibrium assumptions. (a) and (b), depiction of two optima for transition probabilities. The model design for Fc*γ*R IIIb is improved by the assumption of a first-order dependency between alleles. For this purpose, interconnecting transitions and recursion loops are added to the states that emit allele count events. (c), further derivation of the model by assumption of an HNA1a-HNA1c (Na1-SH) tandem allele and irregular repetitions of HNA1c (SH). The graphical representation is as described in Fig. 3.

To gain further insight into the nature of the disequilibrium in the distribution of Fc*γ*R IIIb genotypes, we derived the model shown in Fig. 3e. In the extended model (Fig. 4a and b), we assumed a first-order dependency between different allelic variants. We found this model to be superior to the first model, although at the cost of more parameters (Bayes factor against first model: 2.46 × 10^−21^). The posterior distribution of transition probabilities appeared to be bi-modal (Fig. 4a and b, Suppl. Fig. 4) and revealed a connection between HNA1c (SH) and HNA1A (Na1). The bi-modality of the posterior distribution was expected because of the ambiguity in the order of alleles in the tandem allele, which is either HNA1c (SH) followed by HNA1A (Na1) or vice versa. This connection is compatible with a tandem allele for Fc*γ*R IIIb. The existence of such a duplication composed of HNA1a and HNA1c has been hypothesized previously by a genotyping study for the northern German population^20^. The extended model further indicates a tendency of the HNA1c (SH) allele to be repeated multiple times, which we considered as an artifact of the MLPA method.

We incorporated the above interpretations into a third model that included the HNA1c-HNA1a (SH-Na1) tandem as an additional allele (Fig. 4c). The marginal likelihood of this model was nearly the same as for the second model, further supporting the possible existence of this tandem allele. Moreover, in this third model, the number of free parameters could be reduced from 13 to 7.

For our data set with 387 individuals, the average running time of the MCMC sampler for the models described in Fig. 4a, b and c were about 75–80 seconds for 1000 sampling cycles.

### Resolution of phases for individual copies of Fc*γ*R IIb and IIc genes

The MLPA system determines three polymorphisms for both Fc*γ*R IIb and IIc, genes with opposite functions; Fc*γ*R IIb is an inhibitory receptor and Fc*γ*R IIc an activatory receptor, but the 5′ part of DNA sequences of both receptor genes, including the promoter regions and a gross part of coding sequences, are identical. In consequence, it is not straightforward to separately determine the G-386C and the A-120T SNPs of Fc*γ*R IIb and IIc promoters (compare Fig. 1). Because these promoter polymorphisms have been experimentally shown to influence the expression of their genes^21^, it is important to determine which promoter alleles are located in Fc*γ*R IIb and which are located in Fc*γ*R IIc.

Our approach here was to exploit possible linkages of promoter polymorphisms with two additional polymorphisms determined by the MLPA system, i.e., the functionally relevant I232T polymorphism of Fc*γ*R IIb and the above-described *ORF/Stop codon* polymorphism of Fc*γ*R IIc (Fig. 1). The fact that frequencies of promoter alleles are reported to differ between Fc*γ*R IIb and Fc*γ*R IIc^22^ may also be exploited to resolve the phases of promoter polymorphisms.

**Figure 5:**
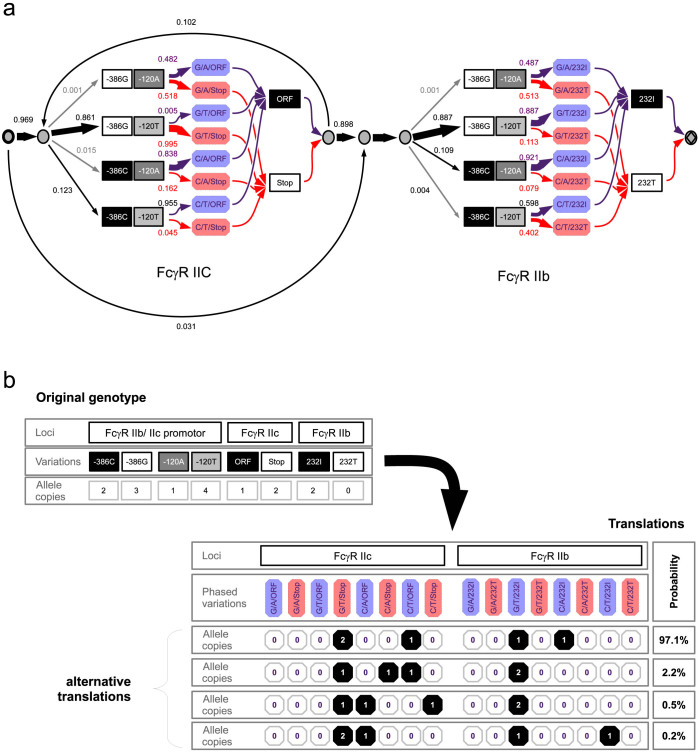
Resolution of haplotype phases of Fc*γ*R IIb and IIc by the Markov model approach. (a), design and transition probabilities of an HMM for two loci with several copy numbers and several allels that takes non-phased MLPA genotypes as input and translates them into phased genotypes. The HMM design reflects this ambiguity between the promoter polymorphisms of Fc*γ*R IIb and IIc by using two separate states emitting the same counting events for these promoter polymorphisms. To introduce translation capability, this model can be used with an alternative set of emissions, depicted here with additional colored octagonal boxes (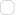) that are equivalent to phased haplotypes. Thus, a single Markov path through this model can emit the original (half-tone square boxes 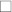) or alternative counting events. Loops were included for Fc*γ*R IIc only, as Fc*γ*R IIb does not exhibit copy number variation. The graphical representation is as described in Fig. 3. (b), non-phased genotypes (**Original genotype**) with translated phased genotypes (**Translations**) and the probabilities of these (Probability).

The corresponding HMM is described in Fig. 5a. In this model, we added an extension to translate non-phased genotypes, as provided from MLPA readouts, into phased genotypes with correctly determined promoter alleles for both receptors (Fig. 5b). For this purpose, the HMM is associated with two alternative emission sets and matrices: one for the non-phased genotypes and the other for phased genotypes. We first inferred transition probabilities for the given input model using non-phased emissions. The expectation value of the transition probabilities' posterior distribution was then used to simulate a very large set of Markov paths. Those Markov paths matching a given non-phased genotype were then used with the alternative emission set. In this setting, a single non-phased genotype may be associated with multiple phased genotypes, the probability of which is also calculated (Fig. 5b). The translated phased genotypes are available for downstream analyses.

In the case of Fc*γ*R IIb and IIc phases, the resulting HMM nearly allows one-to-one translation of genotypes, i.e., one phased genotype with at least 95% probability. Sampling quality was checked by comparison of multiple separate sampler runs, which indicated a sufficient mixing and a good convergence of parameter estimates as well as low autocorrelation times.

Moreover, the frequencies for Fc*γ*R IIc and IIb promoter haplotypes, which are reflected by the inferred transition probabilities (Fig. 5a), are identical to those reported using a long-range PCR method^22^. Of interest, the Fc*γ*R IIc ORF/Stop variation, which determines whether this gene is expressed as a functional receptor on the surface of immune cells^16^, is strongly linked to the -386C/-120T promoter variant. For Fc*γ*R IIb, no relevant linkage was detected between the I232T polymorphism and the promoter haplotypes.

For our data set with 387 individuals, the average running time of the MCMC sampler for the above described model was about 835 seconds for 1000 sampling cycles.

### Dissection of copy number blocks of Fc*γ* receptors IIIa, IIc and IIIb

The Fc*γ*R IIIa, IIc and IIIb form a block on chromosome 1q23.3 that varies in copy numbers. This block is flanked by Fc*γ*R IIa downstream and IIb upstream, with neither gene showing variation in copy numbers. The gene order presented in the consensus map^23^ does not reflect how the gene sequence might look in the case of a copy-number variation.

**Figure 6:**
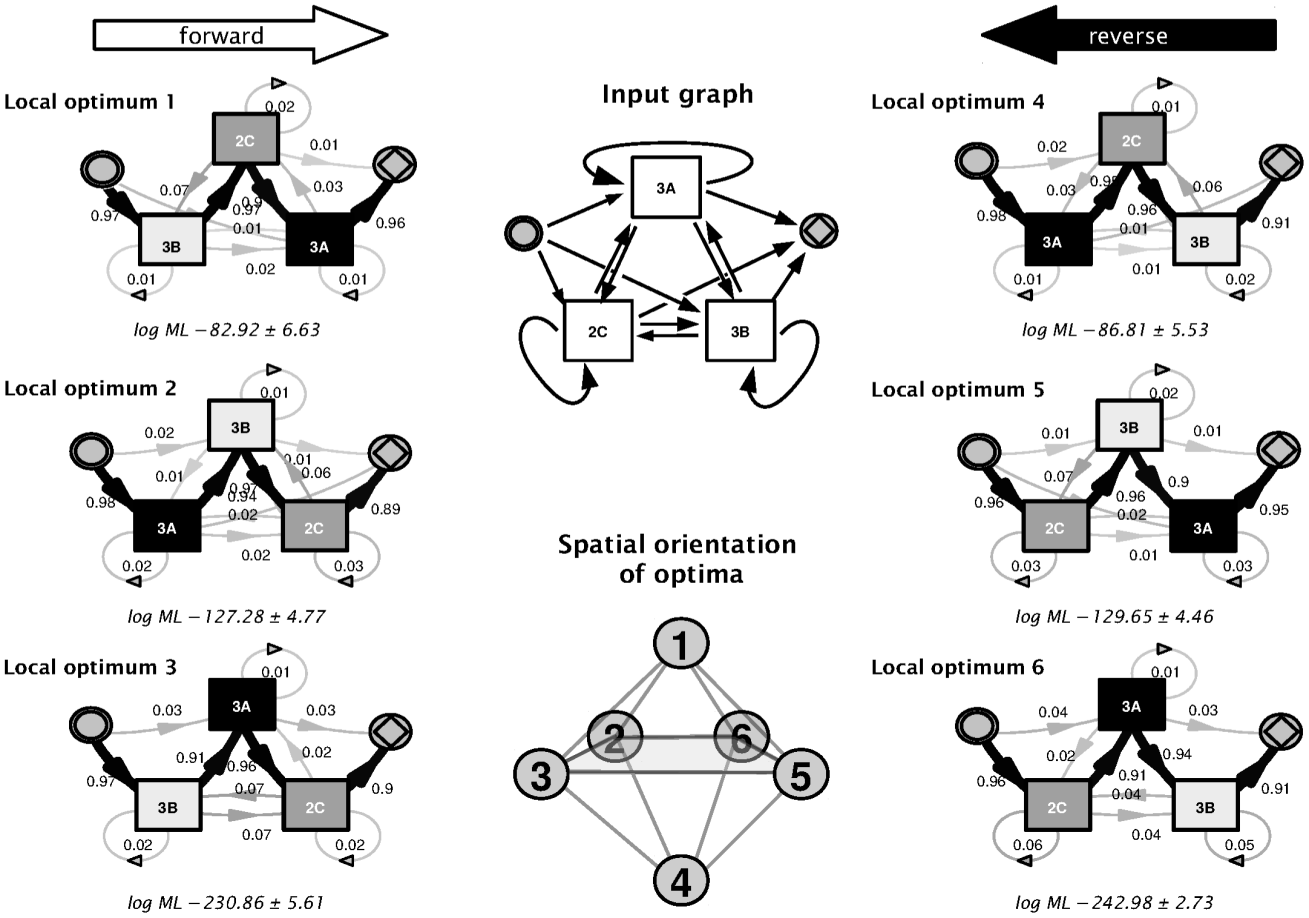
Linkage of Fc*γ*R IIIa, IIc and IIIb copy number variations. To dissect a possible relationship between gains and losses between Fc*γ*R genes, a model graph incorporating all possible connections between the states (**input graph**) was used for inference. Given that genotyping data do not explicitly contain ordering information, and given that the gross of haplotypes carry only one copy of each gene, the highest-probability path through this model orders the three genes Fc*γ*R IIIa (3A), Fc*γ*R IIc (2C) and Fc*γ*R IIIb (3B) in one of 6 different ways; the corresponding optima of transition matrices are spatially oriented in parameter space near the 6 corners of an octahedron. Thus, the samples from 50 separate MCMC simulation runs (compare Suppl. Fig. 5) can be divided by k-means clustering into 6 clusters, which are located near the corner of the octahedron to which they are converging. **Local optimum 1–6**, transition graph corresponding to the average of MCMC transition matrix samples at each of the clusters. Graph edges with transition probability below 0.01 are omitted for clarity. **log ML**, average ± std. dev. of calculated log marginal likelihoods. The graphical representation is as described in Fig. 3.

As a further challenge for the HMM approach, we wanted to determine the chromosomal order of genes in cases of duplications and deletions and reveal connection patterns in copy number variations. For this purpose, we defined a model graph in which all states emitting a counting event are connected in both directions (Fig. 6). This concept inevitably leads to a multimodal posterior distribution, with a posterior distribution that contains more than one sharply peaked high-posterior density island. The most frequent haplotype is expected to harbor one copy of each receptor gene. In the HMM, three genes may be ordered in six different ways along the highest probability path through the HMM, which can be regarded as forward and reverse variants of three unique orders. Thus, we expect at least six main optima for the transition probability matrix, with three symmetry pairs of equivalent likelihoods, as the genotype data set itself does not contain information about the order of genes. If there is complete independence between the copy numbers of individual genes, all optima will have the same likelihood. However, if two genes have a tendency to be duplicated or deleted together, they should be sorted into a neighborhood in the highest-probability Markov path. Practically, this order should be the same — or its reverse — as the order in the consensus gene map. The symmetry between modes is broken if there is any dependence between copy number gains or losses of two genes. Because of the Markov property, a model in which the highest-probability path orders the genes in a way that dependent genes are arranged in sequential neighborhood should be more optimal than other models.

We expected a sharply peaked posterior distribution of transition matrix parameters, with all peaks located distant to each other within the parameter space. Therefore, the MCMC sampler is not very likely to jump between local optima within a finite time scale. To determine a global optimum, we repetitively restarted the MCMC sampler until it converged to use these local optima as *candidate optima*. Local optima were compared using the marginal likelihood calculated by the CJ method. The trace plots visualized in Suppl. Fig. 5 indicate that the MCMC traces converge toward these six expected optima, which are arranged at the corners of an octahedron (compare Fig. 6). The trace plots (Suppl. Fig. 5) furthermore suggest that the 50 runs are sufficient to rank optima. To associate each run with one of the six optima, the samples (after removal of burn-in samples) of all runs were combined and subjected to a *k*-means cluster analysis (with k = 6). The mean of each cluster and its respective marginal likelihood are presented in Fig. 6.

The highest probability path through the HMM for clusters 1 and 2 show the same gene order as the GRCh38 consensus map (in forward and reverse). This pattern suggests that duplicated genes are typically located in a neighborhood and that gene deletions occur in a block-wise fashion. In the case of Fc*γ* receptor genes, Fc*γ*R IIc and Fc*γ*R IIIb form a block that is either deleted or duplicated as one, whereas Fc*γ*R IIIa is more independent from the other two. This finding supports a higher restriction in copy-number variation for this receptor, which is also noticeable in the model shown in Fig. 3c, indicating a lower probability of deletion and duplication than for the other two receptor genes (Fig. 3d and e).

For our data set with 387 individuals, the average running time for 1000 sampling cycles of the MCMC sampler was about 55 seconds for the model described in Fig. 6.

## Discussion

The combined investigation of CNAVs introduces a new level of complexity into genetics, which raises the need of novel tools for analysis and data exploration. These tools should aid the human researcher in uncovering patterns and metastructures that are hidden within data sets.

Challenged with a genetic data set of high complexity, we developed a framework to formulate and evaluate models that allow deeper insights into the nature of copy number and allelic variation.

The original goal of this algorithm was to analyze the output of a certain MLPA system that was developed to determine the CNAV of the highly polymorphic Fc*γ* receptor gene locus^16^,^17^. Differently from other methods for determination of copy number variation, this system uses a redundant probe set, that allows precise and allele-specific differentiation between 0 and 5 copies, e.g. of Fc*γ*R IIIb. This type and quality of data is typically not provided by routine genotyping methods such as TaqMan-based techniques or array-comparative genomic hybridization (array-CGH), which is further outlined by Van Loo *et al.*^24^ in the description of their bioinformatic approach for allele-specific copy number analysis of tumors (ASCAT). However, genotyping data of similar quality and complexity might be provided in the future by special applications of next generation sequencing^25^.

Trying to simply enumerate CNAV genotypes of a population sample to determine their respective frequencies leads to the fundamental *zero-frequency* problem: not all theoretically possible genotypes are found in a population sample. Moreover, any restriction in the virtually infinite number of theoretically possible genotypes is completely arbitrary.

Several approaches have been described to infer haplotype phases in regions with CNAV, such as MOCSphaser^26^, CNVphaser^27^ and polyHap^28^. These approaches either treat copy number variation of genes as extra alleles, residing on two chromosomes (non-internal phasing), or they assume multiple *pseudo*-chromosomes harboring single copies of a gene. By this principle, the chromosomal order of genes and SNPs in CNAV is given by the consensus map. Depending on the complexity of represented genotypes, both methods require a large number of model parameters, and neither avoids the *zero-frequency* problem. MOCSphaser tries to exploit linkage between CNV and neighboring SNP variation, but does not analyse SNPs within genes varying in copy numbers^26^. CNVphaser uses internal phasing and combines an expectation-maximization (EM) with a partition-ligation (PL) algorithm to calculate the frequencies of CNAV haplotypes in a population. To overcome unfavorable properties of the EM algorithm, CNAV haplotypes that are determined to be rare are dropped from inference. Furthermore, CNVphaser is not appropriate for representing multiple genes with different copy numbers by design^27^. polyHap combines both internal and non-internal phasing and uses a (restricted) list of pre-calculated genotypes for inference^28^.

The above-described systems did not match the properties of our MLPA data for the Fc*γ*R gene locus. CNVphaser would not allow the description of CNAVs with different copy numbers, and polyHap could not resolve the ambiguities of Fc*γ*R IIb and IIc promoter polymorphisms. These shortcomings led us to the development of our HMM approach. To analyze complex genotypes, we defined them as the static result of a dynamic process. This process is described by a Markov model that emits events that are summarized as counts of gene alleles. These summarized event counts are equivalent to data from genotyping methods that determine the abundance of local sequence motifs, such as MLPA.

The most remarkable feature of these Markov models, in contrast to the other approaches developed for CNAV, is the incorporation of recursion loops. This functionality leads to a huge reduction in parameters for the model, which remains able to represent all the virtually infinite number of possible genotypes. The introduction of loops is an extension of the Hardy-Weinberg equilibrium. The original Hardy-Weinberg equilibrium assumes independence between chromosomes and a constant frequency of alleles in a population. Using the Markov model approach, we further assume independence between individual gene copies and between copy events. Moreover, we assume that the processes that lead to changes in copy-number variation have reached a steady-state equilibrium. This extended Hardy-Weinberg equilibrium concept is supported by the superior performance of the corresponding HMM. Nevertheless, as we demonstrate with our data set for Fc*γ*R IIIb, the design of HMMs enables an even more elaborate representation of different types of linkage disequilibria, including LOH signatures of certain CNAV generating mechanisms. Because of this simplicity, the current implementation of the algorithm always assumes independence between chromosomes.

With the HMM approach, the user pre-defines a directed model graph, for which the transition probabilities are inferred using Bayesian methodology. For this purpose, we developed a novel hybrid MCMC algorithm that efficiently samples Markov paths along the model, which are then used to simulate realizations of transition probabilities. This sampler not only provides point estimators for transition probabilities, as an expectation-maximization algorithm would do, but also delivers corresponding posterior distributions and credibility intervals. The Markov paths can be considered as a proxy for the true linear chromosomal sequence, containing resolved haplotype phases for individual gene copies. Because of the distinctive intergenic homology of Fc*γ*R genes, the correct assignment of SNPs to the respective promoters of Fc*γ*R IIb and IIc is very difficult. With an extension of HMMs, we were able to translate non-phased haplotypes into phased haplotypes, which were then available for down-stream analyses. The frequency of phased haplotypes for both receptors is equivalent to that reported by Su et al.^22^, further supporting the validity of our HMM approach.

Depending on the HMM graph design, the resulting posterior distribution is multimodal. To cope with this condition, we applied the MCMC sampler repetitively and selected the best result according to a post hoc score. This strategy was used to evaluate the linkage between copy numbers of Fc*γ* receptors IIIa, IIc and IIIb. Although the MLPA data do not contain concrete positional information, a certain order of genes by the HMM transition probabilities seemed to be optimal to describe the genotyping data. Remarkably, this order was identical to the order in the GRCh38 consensus map.

The examples we provide in this study demonstrate how our HMM approach can be applied, adapted and extended for different analytical and statistical tasks. This approach provides a novel methodology, including a novel extension of the Hardy-Weinberg equilibrium concept, to gain insight into complex but important gene regions.

## Methods

### Infrastructure

The software was developed under Linux (Debian^29^ distribution) with R software version 3.0^30^ with packages *MCMCpack*, *coda* and *cluster*, *Rcpp* and *RcppArmadillo* version 0.4.100.2.1 (Edelbuettel, D., RcppArmadillo (Date of access:03/11/2014), http://dirk.eddelbuettel.com/code/rcpp.armadillo.html) and the Boost C++ libraries version 1.53 (Boost Community, Boost.org (Date of access:03/11/2014), http://www.boost.org/). All calculations were performed on a desktop computer with an Intel Core i5 quad-core CPU 660 @ 3,33 GHz (Intel, Santa Clara, CA, USA) with 8 GB RAM.

### Data sets

Genotyping data on healthy German control individuals (*N* = 387) were determined with the FCGR-P110 and FCGR-P111 MLPA kits (MRC Holland, Amsterdam, The Netherlands) according to the manufacturer's instructions. Briefly, 50 ng of genomic DNA was hybridized with twin probes overnight, followed by a ligation reaction. The ligated probes were amplified by PCR, and the size and quantity of the resulting PCR fragments were analyzed with a Beckman CEQ-880 capillary sequencer (Beckman Coulter, Krefeld, Germany). The fragment analysis was performed as recommended by the manufacturer. This data set contains the copy numbers for the different alleles of each Fc*γ*R gene, as outlined in Fig. 1 and further specified in the Suppl. Materials.

The samples for the study were collected from healthy blood donors of the Dept. of Transfusion Medicine of the university hospital of Lübeck. Further samples were provided by *popgen* biological materials collection (Lieb W., *popgen* — Gesundheit für Generationen (Date of access:03/11/2014), http://www.popgen.de/)^31^. All studies with human materials followed the ethical principles established by the Declaration of Helsinki and were approved by the local ethics committee (AZ 10-026 and AZ 08-156). All sample donors gave informed consent.

### Hidden Markov models and graphs

For inference on HMMs, the user provides a directed graph defining valid transitions between states, including one starting and one final state. This graph is walked randomly, once to generate a haplotype and twice for a complete genotype. Haplotypes and genotypes are summaries of counting events that may be emitted during the walking process. The HMMs are described in detail in the Suppl. Materials section.

### Sampling of transition probabilities

Given a model graph, data-augmented Metropolis-within-Gibbs sampling is used to determine the posterior distribution of transition probabilities. The Markov path *pairs* compatible with observed genotypes are the latent variables in the sampling process. The sampler therefore cycles between the determination of Markov paths and transition probabilities.

Given Markov path *pairs* along the model, a realization of the transition probability matrix can be simulated using a *MatrixDirichlet* distribution (Suppl. Materials) as a conjugate prior. To simulate Markov path *pairs*, a hybrid approach was used that provided the highest sampling efficiency. The first component of this hybrid approach is an *incomplete* Gibbs sampler. For this process, an empirically defined number *n* of random Markov path *pairs* is produced, given the transition probabilities matrix *A*. Markov paths compatible with a single genotype of the observations are taken as a new proposal of the respective individual, and incompatible Markov path *pairs* are dropped. If no proposal is found for an individual, the identity kernel is used as a default. As with a normal Gibbs sampler, the acceptance rate for the proposal is 1. However, if *A* is not near to an optimum, the fraction of individuals for which the identity proposal is used is so high that the sampler does not move efficiently.

Therefore, the second component of the hybrid approach is used as a backup. This backup is provided by a balanced recursive tree search, termed the squirrel algorithm, which defines a symmetric MCMC transition kernel for a random walk. The sampling process is further described in detail in the Suppl. Materials.

### Calculation of the marginal likelihood

During sampling, both latent Markov paths and transition probabilities are recorded and used to calculate the marginal likelihood for a two-block sampler, as described by Chib and Jeliazkov^18^. The Chib method requires a likelihood function, which is by itself computationally intractable here. To approximate the likelihood function, the frequency of observed genotypes is calculated from a large number of samples generated for given transition probabilities. The naïve enumeration model (NEM) uses Jeffrey's prior to determine a Dirichlet distribution of genotype frequencies. Genotypes that were observed at least once in the data set were used as categories, plus one category for unobserved genotypes. The parameters for the posterior Dirichlet distribution were calculated from the observation counts for each category, enabling the exact calculation of the marginal likelihood. Because of the arbitrary restriction of categories, the marginal likelihood tends to become more overestimated the more complex the genotype observations become. Nevertheless, the NEM is useful for evaluating and comparing the performance of an HMM.

### URLs

Our implementation of the algorithm including the genetic control data set described in this article is available as a source package CNAV for R at http://r-forge.r-project.org/projects/cnav/.

## Author Contributions

A.R. designed research, performed experiments, analyzed data, wrote the software package and the manuscript. K.G.R. reviewed analyses and figures and wrote the manuscript. S.I. wrote the manuscript. S.M. wrote the software package, reviewed analyses and figures and wrote the manuscript. R.V. reviewed analyses and figures and wrote the manuscript.

## Supplementary Material

Supplementary Information Supplementary Materials
