# Supplementary material for: Hardy-Weinberg equilibrium revisited for inferences on genotypes featuring allele and copy-number variations
Source: Sci Rep. 2015 Mar 13;5:9066. doi: 10.1038/srep09066 (PMC4357990; doi:10.1038/srep09066)
Supplement: Supplementary Information — Supplementary Materials [file srep09066-s1.pdf]

Hardy-Weinberg equilibrium revisited for inferences on genotypes featuring allele and copy-number variations

Supplementary Materials

Recke, Andreas   Recke, Klaus-Günther   Ibrahim, Saleh
Möller, Steffen   Vonthein, Reinhard

December 10, 2014

Supplementary Materials

Details of the data set

The implementation of CNAV is completely agnostic to the technology that delivered the data. At the time of writing, the most common technologies used to distinguish alleles of genes (sequence variation) and their genetic abundance (copy numbers) are multiplex ligation-dependent probe amplification (MLPA) and next-generation sequencing (NGS).

To use the data determined by MLPA or NGS in our approach, we first established an appropriate representation. To support an unambiguous integration of CNAV in novel projects, this document outlines the input and output formats as well as the core principles of the algorithm.

We describe a gene with l different alleles by an l-vector of the numbers of these alleles present, $\mathbf{g} = (g_1, g_2, \ldots, g_l)$, $\mathbf{g} \in \mathbb{N}_0^l$. The three genotypes of a biallelic gene without CNV would be $(2, 0)$, $(1, 1)$, and $(0, 2)$. The more alleles, the longer the vector. Without CNV, its elements sum to 2. $(2, 2, 0)$ describes a genotype with two copies of a gene with three alleles, one of which was not observed in this case.

The haplotypes of such a gene are described by just such a vector, the sum of its elements being the copy number. The vector describing a genotype is the sum of two vectors describing haplotypes such as $(2, 2, 0) = (1, 1, 0) + (1, 1, 0)$ or $(2, 2, 0) = (2, 0, 0) + (0, 2, 0)$.

Now consider k genes and denote genotypes and haplotypes as k-vectors of vectors. Still, one genotype $\mathbf{G} = (\mathbf{g}_1, \ldots, \mathbf{g}_k)$ is the element-wise sum of two haplotypes residing on the two chromosomes of a complete genome: $\mathbf{G} = \mathbf{H}_1 + \mathbf{H}_2$. All $\mathbf{G}$, $\mathbf{H}_1$, and $\mathbf{H}_2$ are elements of set $\mathcal{G}$.

Given a number of M individuals, the complete genotype of a single individual i is $\mathbf{G}_i$. The data

from the whole cohort are combined into a family $\mathcal{F}$

$$\mathcal{F} = (\mathbf{G}_i)_{i=1...M} \tag{1}$$

The above definitions support the tabular representation of all genotype data (Suppl. Tab. 1).

Introduction to Hidden Markov Models (HMMs)

Hidden Markov models describe stochastic sequences in a comprehensive way. These models are widely used for BLAST sequence comparison and inference of haplotypes in genetic association studies, e.g., by HaploView [30]. We derive these HMMs to represent not only haplotypes with allelic variation but also those with copy-number variation.

Our approach allows the researcher to design the Markov model graph for which transition probabilities are inferred to match genotype data that are determined e.g., by MLPA.

An HMM λ is defined as a quintuple $\lambda = (S, V, A, B, \pi)$, containing the following components:

- the set of hidden states $S = \{s_1, \dots, s_n\}$;
- the alphabet of emissions $V = \{\mathbf{v}_1, \dots, \mathbf{v}_m\}$, $\mathbf{v}_1, \dots, \mathbf{v}_m \in \mathcal{G}$;
- the transition probability matrix $A = (a_{ij}) \in \mathbb{R}^{n \times n}$, with a_{ij} being the probability of a transition from state s_i to state s_j;
- the probabilities of observed emissions $B = (b_{ij}) \in \mathbb{R}^{n \times m}$, with b_{ij} being the probability in state s_i for an emission $\mathbf{v}_j$. By convention, for each i, the probability is 1 for exactly one b_{ij}; a hidden state is defined by the emission of the neutral element $\mathcal{O}$;
- and the starting probabilities $\pi \in \mathbb{R}^n$.

By convention, the starting state of an HMM is always s_1 (source) and the final state is always s_n (sink). Thus, for initial state probabilities, we set $\pi = (1, 0, 0, \dots, 0)$. Each Markov path $\mathbf{X} = (\mathbf{x}_t)_{t=1,\dots,\mathbf{x}_T}$ is a sequence of T subsequent states, with $\mathbf{x}_t \in S$, emits a series of events $\mathbf{Y} = (\mathbf{y}_t)_{t=1,\dots,\mathbf{x}_T}$, with $\mathbf{y}_t \in V$.

Let $\mathcal{X}$ be the set of all possible Markov paths through a given HMM λ.

A function f that maps a single Markov path $\mathbf{X} \in \mathcal{X}$ to its associated haplotype $\mathbf{H} \in \mathcal{G}$:

$$f : \mathcal{X} \to \mathcal{G},\ \mathbf{X} \mapsto \mathbf{H} = f(\mathbf{X}) = \sum_{t=1}^{T} \mathbf{y}_t = \mathbf{y}_1 + \mathbf{y}_2 + \dots + \mathbf{y}_T \tag{2}$$

Because the emission probabilities are all 1 by convention, the term *hidden* here applies to the fact that the exact sequence of states, i.e., the Markov path $\mathbf{X}$, and emissions $\mathbf{Y}$ is hidden, and only the result of the association function $f(\mathbf{X})$ is visible. Moreover, this function is not injective, allowing one haplotype $\mathbf{H}$ to be associated with multiple Markov paths. This possibility has interesting consequences for the distribution of genotypes, which are depicted in Suppl. Tabs. 2 and 3.

A complete genotype $\mathbf{G}$ consists of two haplotypes $\mathbf{H}_1$ and $\mathbf{H}_2$. Thus, we associate a complete genotype with a pair $(\mathbf{X}_1, \mathbf{X}_2) \in \mathcal{X}^2 = \mathcal{X} \times \mathcal{X}$ of Markov paths:

$$f : \mathcal{X}^2 \to \mathcal{G},\ (\mathbf{X}_1, \mathbf{X}_2) \mapsto \mathbf{G} \ =\ f(\mathbf{X}_1, \mathbf{X}_2) \ =\ f(\mathbf{X}_1) + f(\mathbf{X}_2) \tag{3}$$

The current implementation of our approach uses the above definition of HMMs. The two haplotypes are assumed to be independent and are treated independently. Nevertheless, this definition may be developed to reflect dependency structures between haplotypes. However, we decided not to extend the above definition here because this would dilute the focus of the current technical report.

Probability of Markov paths

For a given Markov path $\mathbf{X} = (\mathbf{x}_t)_{t=1,\dots,T}$ of length T and defined starting state s_1, the probability $\text{Prob}(\mathbf{X}|A)$ given the transition probability matrix A can be calculated as

$$\text{Prob}(\mathbf{X}|A) \ =\ \prod_{t=2}^{T} \text{Prob}(\mathbf{x}_t|\mathbf{x}_{t-1}, A) \tag{4}$$

As the two Markov paths in a *pair* are independent, the probability of the *pair* $(\mathbf{X}_1, \mathbf{X}_2)$ is

$$\text{Prob}((\mathbf{X}_1, \mathbf{X}_2)|A) \ =\ \text{Prob}(\mathbf{X}_1|A)\,\text{Prob}(\mathbf{X}_2|A). \tag{5}$$

For Bayesian inference of transition matrices, we further seek to identify a matrix representation of Markov paths that can be handled easily. For this purpose, we define a counting matrix as the sufficient statistic $\mathbf{C}(\mathbf{X})$ for any Markov path $\mathbf{X}$:

Definition 1. *The counting matrix* $C = (c_{ij}) = \mathbf{C}(\mathbf{X})$ *summarizes a Markov path* $\mathbf{X}$*, with* c_{ij} *being the count of one-step transitions from* s_i *to* s_j*.*

Given a constant A, and $\text{Prob}(x_t = s_j \,|\, x_{t-1} = s_i, A) \ =:\ a_{ij}$, we can use the associative property and re-write eqn. 4 as

$$\text{Prob}(\mathbf{X}|A) \ =\ \prod_{a_{ij}>0} \prod_{t=2}^{T} a_{ij}^{\mathbf{1}_{\{x_t = s_j \wedge x_{t-1} = s_i\}}} \ =\ \prod_{a_{ij}>0} a_{ij}^{c_{ij}} \tag{6}$$

Using exponentiation rules, the counting matrices of multiple independent Markov paths $\mathbf{X}_1, \mathbf{X}_2, \dots$ can be summarized into one counting matrix $C \ =\ \mathbf{C}(\mathbf{X}_1) \ +\ \mathbf{C}(\mathbf{X}_2) \ +\dots$. The motivation for this cumulative counting matrix is described below under Bayesian inference of the transition matrix.

Likelihood function for single genotypes

Let $\mathcal{X}^2 = \mathcal{X} \times \mathcal{X}$ be the set of all possible Markov path *pairs*; then, the set ${\mathcal{X}^2}_{\mathbf{G}} \subseteq \mathcal{X}^2$ is the subset of all Markov path *pairs* that would produce a genotype $\mathbf{G}$ as the sum of combined emissions.

Definition 2. ${\mathcal{X}^2}_{\mathbf{G}} = \{(\mathbf{X}_1, \mathbf{X}_2) \,|\, (\mathbf{X}_1, \mathbf{X}_2) \in \mathcal{X}^2 \wedge \mathbf{G} = f(\mathbf{X}_1) + f(\mathbf{X}_2)\}$*, with* $f(\mathbf{X})$ *as the association function defined in eq. 3.*

The likelihood function $\text{Prob}(\mathbf{G}|A)$ for a single genotype $\mathbf{G}$ is given as the sum of probabilities of all

alternative Markov paths *pairs* $(\mathbf{X}_1, \mathbf{X}_2) \in \mathcal{X}^2$:

$$\text{Prob}(\mathbf{G}|A) = \sum_{(\mathbf{X}_1,\mathbf{X}_2)\in\mathcal{X}^2{}_{\mathbf{G}}} \text{Prob}(\mathbf{X}_1|A) \cdot \text{Prob}(\mathbf{X}_2|A) \tag{7}$$

Depending on the Markov model design, $\mathcal{X}^2{}_{\mathbf{G}}$ may become so large that this likelihood function becomes computationally intractable. Implementing an expectation maximization algorithm is not practically feasible. Therefore, and because it provides probability distributions for model parameters, we decided to use data-augmented Metropolis-within-Gibbs sampling.

Definition of a conjugate prior for the transition probability matrix

The presented algorithm for Bayesian inference of HMMs takes advantage of the possibility to define an exact distribution function for the transition probability matrix. This functionality allows for the performance of Gibbs sampling of the transition probability matrix A.

Given counts $\mathbf{c}$ of categorical events, which are distributed multinomially, Jeffrey's prior, a Dirichlet distribution $Dirichlet(\alpha)$ with vector parameter $\alpha = (0.5)$, is typically used to calculate the exact posterior distribution of associated categorical probabilities. In the present approach, a Dirichlet distribution is used to calculate the exact posterior distribution of probabilities for one-step transitions from one state of the Markov model to the subsequent states. Thus, we can combine the probability distribution for each state into a joint distribution of independent Dirichlet distributions.

Here, we define such a distribution as MatrixDirichlet, for which we use the matrix parameter Ξ (Def. 3). Each row vector of Ξ is the parameter vector used for one of the Dirichlet distributions.

Definition 3. $\Xi = (\xi_{ij})$*, with* $\xi_{ij} \in \mathbb{R}_{\geq 0}$*.* $\xi_{ij} > 0$*,* if and only if *the corresponding transition is valid.*

In this way, the set of valid Markov model transitions is encoded by the distribution parameter Ξ. Due to the Markov property, the probability distributions for separate states are independent from each other. Thus, we can define the probability density function of the complete transition probability matrix as the product of the probability density functions of each row. However, a standard Dirichlet distribution would not be defined by a parameter vector containing zeros. We hence re-define the Dirichlet density functions for each row A_i of the transition probability matrix A (Def. 4).

Definition 4. $f(A_i; \Xi_i) = f(a_{i1}, \ldots, a_{in}; \xi_{i1}, \ldots, \xi_{in}) = \frac{1}{B(\xi_{i1}, \ldots, \xi_{in})} \cdot \prod_{j,\, \xi_{ij}>0} a_{ij}^{\xi_{ij}-1}$

with $B(\xi_{i1}, \ldots, \xi_{in}) = \frac{\prod_{j,\, \xi_{ij}>0} \Gamma(\xi_{ij})}{\Gamma(\sum_{j,\, \xi_{ij}>0} \xi_{ij})}$.

Together, we define the probability density function for the MatrixDirichlet distribution as Def. 5.

Definition 5. $f\colon \mathbb{R}^{n\times n} \to \mathbb{R}_{\geq 0},\ A \mapsto f(A; \Xi) = \prod_{i=1}^{n} f(A_i; \Xi_i)$ *, with* Ξ *being the matrix parameter and* A *a transition probability matrix.*

This approach allows to specify the user input λ_0 for the HMM inference as in Def. 6.

Definition 6. *The user input for HMM inference* $\lambda_0 = (S, V, \Xi, B, \pi)$*, with* Ξ *as the matrix parameter for the conjugate MatrixDirichlet distribution.*

Bayesian inference of the transition matrix

Let C be the summarized counting matrix representing the family of Markov paths *pair* realizations compatible with a genotype data set $\mathcal{F} = (\mathbf{G}_i)_{i=1,\ldots,N}$ of N individuals. Furthermore, let $\Xi^{(0)}$ be a matrix prior parameter for the MatrixDirichlet distribution of the transition probability matrix A. Then, the posterior distribution $\text{Prob}(A|C)$ can be determined by eqn. 13. This equation is equivalent to an update of the row-wise Dirichlet distributions.

$$\text{Prob}(A|C) \propto \text{Prob}(C|A)\ \text{Prob}(A) \tag{8}$$

$$= \prod_{i,j,\, c_{ij}>0} a_{ij}^{c_{ij}} \cdot \prod_{i=1}^{n} \frac{1}{B(\xi_{i1},\ldots,\xi_{in})} \cdot \prod_{\xi_{ij}>0} a_{ij}^{\xi_{ij}-1} \tag{9}$$

$$\propto \prod_{j,\, \xi_{ij}>0} a_{ij}^{c_{ij}+\xi_{ij}-1} \tag{10}$$

$$\Rightarrow \quad \text{Prob}(A|C) = \prod_{i=1}^{n} \frac{1}{B(c_{i1}+\xi_{i1},\ldots,c_{in}+\xi_{in})} \cdot \prod_{\xi_{ij}>0} a_{ij}^{c_{ij}+\xi_{ij}-1} \tag{11}$$

$$A \sim \text{MatrixDirichlet}(\Xi^{(0)}) \tag{12}$$

$$A\,|\,C \sim \text{MatrixDirichlet}(\Xi^{(0)} + C) \tag{13}$$

Details on sampling of Markov paths

For the sampling of Markov paths with a given transition probability matrix A, we combine two proposal algorithms: a Gibbs-like sampling approach and a random-walk sampler (squirrel algorithm), which is based upon a recursive tree search. Both proposal algorithms have specific strengths and weaknesses that crucially depend on A. In both algorithms, Markov path *pairs* for individual genotypes G_i in the data set $\mathcal{F}$ are sampled separately.

As prior distribution for Markov path *pairs*, we apply a uniform discrete distribution:

$$\text{Prob}((\mathbf{X}_1, \mathbf{X}_2)) \;=\; \frac{1}{|\mathcal{X}^2{}_{G_i}|} \tag{14}$$

with $\mathcal{X}^2{}_{G_i}$ as the set of Markov path *pairs* $(\mathbf{X}_1, \mathbf{X}_2)$ that are compatible with genotype G_i.

Sampling and filtering of random Markov paths - drop-out or incomplete Gibbs sampling

Given a transition probability matrix A, the Markov path *pair* $(\mathbf{X}_1, \mathbf{X}_2)$ associated with each genotype $\mathbf{G}_i$ in the observations can be simulated by a modified Gibbs sampling, which we denote here as *incomplete* Gibbs sampling. The idea is to produce $(\hat{\mathbf{X}}_1, \hat{\mathbf{X}}_2)$ *pairs* of Markov paths by walking the Markov model repeatedly in a random fashion according to the transition probability matrix A until we find an $(\hat{\mathbf{X}}_1, \hat{\mathbf{X}}_2)$ where $f\left((\hat{\mathbf{X}}_1, \hat{\mathbf{X}}_2)\right) = \mathbf{G}_i$.

For practical purposes, this process is stopped after a fixed number n of repetitions, and an identity kernel is used for the proposal: $(\hat{\mathbf{X}}_1, \hat{\mathbf{X}}_2) = (\mathbf{X}_1, \mathbf{X}_2)$. By this mechanism, the general idea of Gibbs sampling is not sacrificed: every proposal including the identity proposal is accepted.

If the transitions probability matrix is nearly optimal, it will produce such a Markov path *pair* with acceptable efficiency. Otherwise, the algorithm might require an unacceptably large number of random samples to provide enough new proposals. To overcome this issue, this algorithm is combined with the below-described squirrel algorithm.

Balanced recursive tree search (squirrel algorithm)

Whereas the above-described approach is very effective when transition probabilities are nearly optimal, it may fail to compute truly new proposals instead of identity proposals for all genotypes in the observations, as the number of random samples is restricted for practical reasons.

For this purpose, new Markov paths fitted to the observations must be determined by a recursive tree search. To use a recursive tree search for a Metropolis-Hastings update, the search algorithm is implemented to obtain a random-walk behavior. The pattern by which the algorithm finds the solutions resembles a squirrel jumping through the branches of a tree (possible Markov paths) looking for nuts (solutions).

This implementation is based on the property of pseudo-random number generators used to always deliver the same series of random numbers given the same random seed r_0. The random search then tries to find a new *pair* of Markov paths $(\hat{\mathbf{X}}_1, \hat{\mathbf{X}}_2) \in \mathcal{X}^2_{\mathbf{G}}$ that produce a genotype $\mathbf{G}$ (see Def. 2). Let r_i be the ith random number that governs the order by which the possible succeeding states of x_i are tested.

For implementation, the *pair* of Markov paths is concatenated, with the final state of $\mathbf{X}_1$ followed by the first state of $\mathbf{X}_2$.

The elements $(\mathbf{X}_1, \mathbf{X}_2)$ are then ordered by the relation $\mathcal{R}_{r_0}$, as defined by Def. 7.

Definition 7. $\mathcal{R}_{r_0} \subset \mathcal{X}^2_{\mathbf{G}} \times \mathcal{X}^2_{\mathbf{G}}$ *is an order of* $\mathcal{X}^2_{\mathbf{G}}$*, which is completely determined by the random seed* r_0.

Based on this order relation $\mathcal{R}_{r_0}$, we can further define $\mathrm{S}^{(\text{-}1)}((\mathbf{X}_1, \mathbf{X}_2))$ as the predecessor and $\mathrm{S}^{(+1)}((\mathbf{X}_1, \mathbf{X}_2))$ as the successor of $(\mathbf{X}_1, \mathbf{X}_2)$.

We now define a Markov kernel $\mathrm{K}_{r_0}((\mathbf{X}_1, \mathbf{X}_2), A)$ with $A = \{\mathrm{S}^{(\text{-}1)}((\mathbf{X}_1, \mathbf{X}_2)), \mathrm{S}^{(+1)}((\mathbf{X}_1, \mathbf{X}_2))\}$ for a given order relation $\mathcal{R}_{r_0}$. If

$$\mathrm{Prob}\left(Z_{t+1} = \mathrm{S}^{(\text{-}1)}((\mathbf{X}_1, \mathbf{X}_2)) \mid Z_t = (\mathbf{X}_1, \mathbf{X}_2)\right) \tag{15}$$

$$= \mathrm{Prob}\left(Z_{t+1} = \mathrm{S}^{(+1)}((\mathbf{X}_1, \mathbf{X}_2)) \mid Z_t = (\mathbf{X}_1, \mathbf{X}_2)\right) \tag{16}$$

$$= 0.5 \tag{17}$$

$$\tag{18}$$

with random variables $Z_t, Z_{t+1} \in \mathcal{X}^2{}_{\mathbf{G}}$, this is in fact a *palindromic* kernel fulfilling the condition of reversibility because of the relation

$$\mathrm{S}^{(+1)}\left(\mathrm{S}^{(\text{-}1)}((\mathbf{X}_1, \mathbf{X}_2))\right) = \mathrm{S}^{(\text{-}1)}\left(\mathrm{S}^{(+1)}((\mathbf{X}_1, \mathbf{X}_2))\right) = (\mathbf{X}_1, \mathbf{X}_2) \tag{19}$$

By choosing the random seed r_0 from a completely arbitrary set, this process defines a state-dependent mixture kernel [31] preserving complete reversibility. Using this kernel and a flat prior distribution for

$(\mathbf{X}_1, \mathbf{X}_2)$, we can calculate the acceptance probability p_A as follows:

$$p_A = \min\left(1; \frac{\text{Prob}((\hat{\mathbf{X}}_1, \hat{\mathbf{X}}_2)|A)}{\text{Prob}((\mathbf{X}_1, \mathbf{X}_2)|A)}\right), \text{ with } (\hat{\mathbf{X}}_1, \hat{\mathbf{X}}_2), (\mathbf{X}_1, \mathbf{X}_2) \in \mathcal{X}^2{}_{\mathbf{G}} \tag{20}$$

Markov chain mixing with this algorithm can be further improved by randomly choosing an n,

$$n \sim \textit{discrete uniform}(1, N), \text{ with } n, N \in \mathbb{N} \tag{21}$$

and proposing the nth predecessor $\text{S}^{(\text{-n})} = \text{S}^{(\text{-1})}(\text{S}^{(\text{-(n-1)})})$ or successor $\text{S}^{(\text{-n})} = \text{S}^{(+1)}(\text{S}^{(+(\text{n-1}))})$, with $\text{S}^{(\text{-1})}$ and $\text{S}^{(+1)}$ as defined above.

The squirrel algorithm is unbiased, but it suffers from the combinatoric complexity of Markov paths. This algorithm therefore may require a large number of cycles until a stationary distribution is achieved. However, it always delivers a proposal for the Markov chain, a property that makes this algorithm ideal for combination with the *incomplete* Gibbs sampling algorithm described above.

Determination of the marginal likelihood of an HMM

To calculate the marginal likelihood $\text{Prob}(\mathcal{F}|\lambda_0)$ of an HMM setting λ_0 (compare 6), we use the method described by Chib and Jeliazkov a two-block Gibbs sampler[18].

Given the observed genotype data $\mathcal{F} = \mathbf{G}_1, \ldots, \mathbf{G}_N$ of N individuals, with $\mathbf{G}_i \in \mathcal{G}$, we choose a transition probability matrix A, preferably at high posterior density. The prior density $\text{Prob}(A)$ is given by

$$A \sim \textit{MatrixDirichlet}(\Xi^{(0)}). \tag{22}$$

The posterior density $\text{Prob}(A \,|\, \mathcal{F})$ is estimated as previously described [18]:

$$\text{Prob}(A \,|\, \mathcal{F}) \;=\; U^{-1} \sum_{u=1}^{T} \text{Prob}(A \,|\, \Xi^{(u)}), \text{with} \tag{23}$$

$$\Xi^{(u)} = \Xi^{(0)} + C^{(u)}, \text{and} \tag{24}$$

$$A|\Xi^{(u)} \sim \textit{MatrixDirichlet}(\Xi^{(u)}). \tag{25}$$

$C^{(1)}, \ldots, C^{(U)}$ is the sampled series of counting matrices (compare *Bayesian inference of the transition matrix*). Because of the computational intractability of the likelihood function, we estimate its value using an approximation by simulating a large number n of Markov path pairs, resulting in the simulated genotype data set $\boldsymbol{\Upsilon}^*$, with $n = |\boldsymbol{\Upsilon}^*|$.

Let $\mathcal{G}^{\mathcal{F}}$ be the set of *different* genotypes of the data set:

$$\mathcal{G}^{\mathcal{F}} = \{\mathbf{G} \,|\, \mathbf{G} \in \mathcal{F}\}, \tag{26}$$

$$\text{with } |\mathcal{G}^{\mathcal{F}}| \text{ being the cardinality of } \mathcal{G}^{\mathcal{F}} \tag{27}$$

Then, taking 0.5 as initial pseudo-counts for each genotype category (compare Jeffrey's prior), the

probability $\text{Prob}(\mathbf{G})$ of each genotype $\mathbf{G} \in \mathcal{G}^{\mathcal{F}}$ in the observed data set is estimated as

$$\text{Prob}(\mathbf{G}) \doteq \frac{0.5 + \sum\limits_{\mathbf{G}' \in \mathcal{G}^{\boldsymbol{\Upsilon}^*}} \mathbf{1}_{\{\mathbf{G}=\mathbf{G}'\}}}{0.5\,(1 + |\mathcal{G}^{\mathcal{F}}|\,) + |\boldsymbol{\Upsilon}^*|}, \tag{28}$$

with $|\boldsymbol{\Upsilon}^*| \to \infty$.

The likelihood function $f(\mathcal{F}|A)$ is a probability mass function of the corresponding multinomial distribution (eq. 31), with x_i being the number of occurrences of genotype $\mathbf{G}_i$ in the observed data $\mathcal{F}$, and $p_i = \text{Prob}(\mathbf{G}_i)$

$$x_i = \sum_{\mathbf{G}' \in \mathcal{G}^{\mathcal{F}}} \mathbf{1}_{\{\mathbf{G}_i=\mathbf{G}'\}} \tag{29}$$

$$n = 1 + \sum_{i=0}^{k} x_i \tag{30}$$

$$f(\mathcal{F}|A) = f(x_1, x_2, \ldots, x_k; n; p_1, p_2, \ldots, p_k) = \frac{n!}{1!x_1!x_2!\ldots x_k!} \cdot \sum_{i=1}^{k} {p_i}^{x_i} \text{ , with } k = |\mathcal{F}| \tag{31}$$

A value 1 is added here to the cardinality of $\mathcal{G}^{\mathcal{F}}$ to represent a category of elements $\mathbf{G} \in \boldsymbol{\Upsilon}^* \backslash \mathcal{F}$, that are found in the simulated data set but not in the observations. Then, the marginal likelihood is calculated as described [18]:

$$\text{Prob}(\mathcal{F}|\lambda) = \frac{f(\mathcal{F}|A)\ \text{Prob}(A)}{\text{Prob}(A|\mathcal{F})} \tag{32}$$

Determination of marginal likelihood of an NEM

The marginal likelihood of each HMM setting is routinely compared to the marginal likelihood of a NEM $M^{(naive)}$. The posterior distribution of probabilities $p_i = \text{Prob}(\mathbf{G}_i|\mathcal{G})$ of each genotype $\mathbf{G}_i$ can be described analytically using a conjugate Dirichlet prior (eq. 33).

$$(p_0, p_1, p_2, \ldots, p_k) \sim Dir(\alpha + (x_0, x_1, x_2, \ldots, x_k)), \tag{33}$$

using the same counting variables x_i for genotypes $\mathbf{G}_i$ as in eq. 29 and $x_0 := 0$ as a surrogate for unobserved genotypes.

$\alpha = (\alpha_0, \alpha_1, \ldots, \alpha_k) = (0.5, \ldots, 0.5)$ is the parameter for the prior distribution.

The marginal likelihood $\text{Prob}(\mathcal{G} \mid M^{(naive)})$ is then determined as in eq. 35.

$$B(\mathbf{x}^*) = \frac{\prod\limits_{i=1}^{k} \Gamma(x_i^*)}{\Gamma\left(\sum\limits_{i=1}^{k} x_i^*\right)} \tag{34}$$

$$\text{Prob}(\mathcal{G} \mid M^{(naive)}) = \frac{\frac{1}{B(\mathbf{x}+1)} \prod\limits_{i=0}^{k} p_i^{x_i} \cdot \frac{1}{B(\alpha)} \prod\limits_{i=0}^{k} p_i^{\alpha_i - 1}}{\frac{1}{B(\mathbf{x}+\alpha)} \prod\limits_{i=0}^{k} p_i^{x_i + \alpha_i - 1}} = \frac{\frac{1}{B(\mathbf{x}+1)} \cdot \frac{1}{B(\alpha)}}{\frac{1}{B(\mathbf{x}+\alpha)}} \tag{35}$$

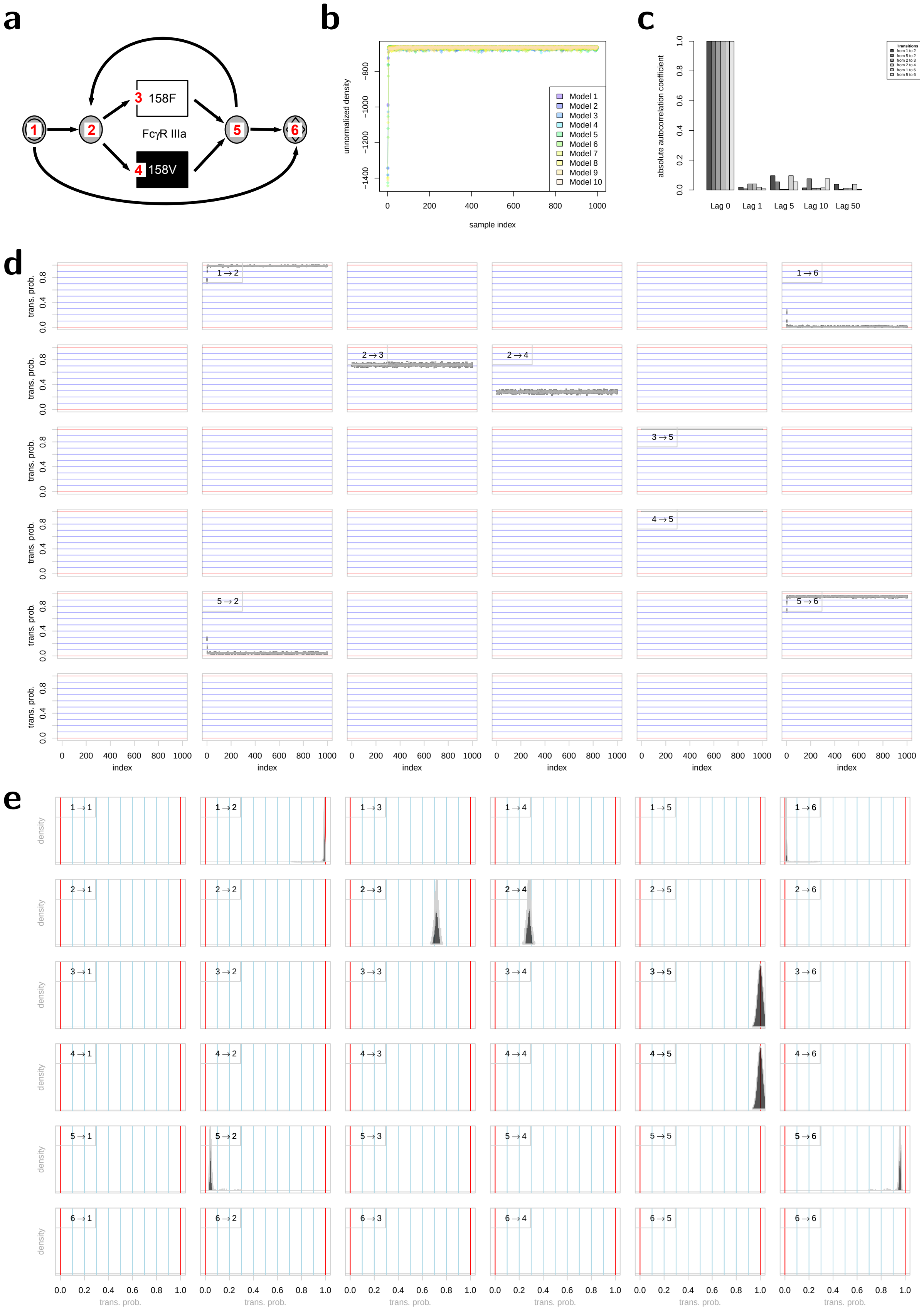

SUPPL. FIG. 1 – **Analysis of sampler output for basic HMM for FcγR IIIA.** **a**, state numbering scheme of the HMM graph depicted in Fig. 3c. **b**, trace of non-normalized density values of 10 separate sampler runs. **c**, autocorrelation plot of a single sampler run for each varying transition probability. **d**, trace plot of transition probabilities from 10 separate sampler runs. The plots are oriented according to the transition matrix, with each row indicating the first state and each column the second state of a one-step transition. **e**, corresponding density plots of transition probabilities using a Gaussian kernel estimator for each marginalized parameter.

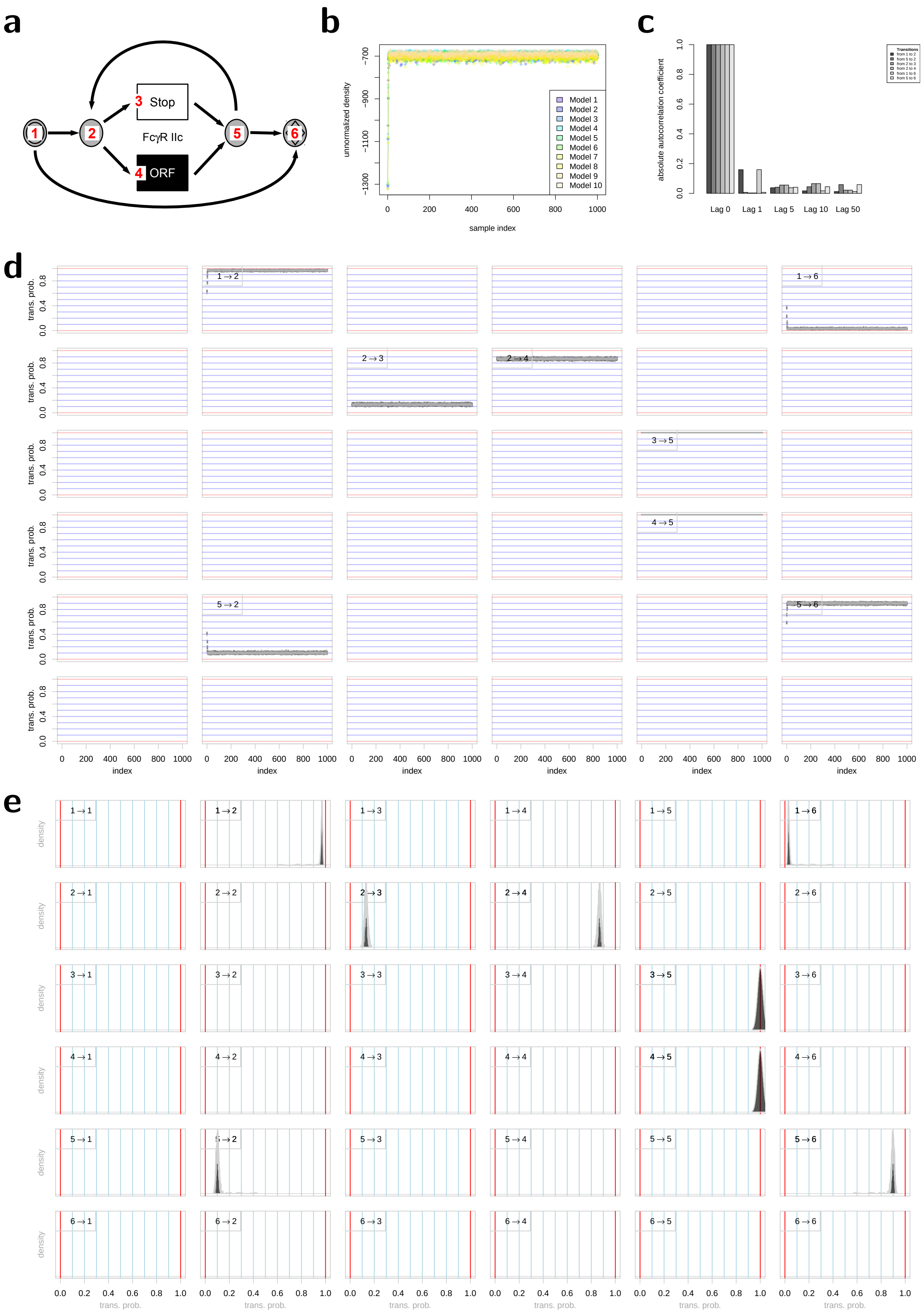

Suppl. Fig. 2 – **Analysis of sampler output for basic HMM for FcγR IIc.** **a**, state numbering scheme of the HMM graph depicted in Fig. 3d. **b**, trace of non-normalized density values of 10 separate sampler runs. **c**, autocorrelation plot of a single sampler run for each varying transition probability. **d**, trace plot of transition probabilities from 10 separate sampler runs. The plots are oriented according to the transition matrix, with each row indicating the first state and each column the second state of a one-step transition. **e**, corresponding density plots of transition probabilities using a Gaussian kernel estimator for each marginalized parameter.

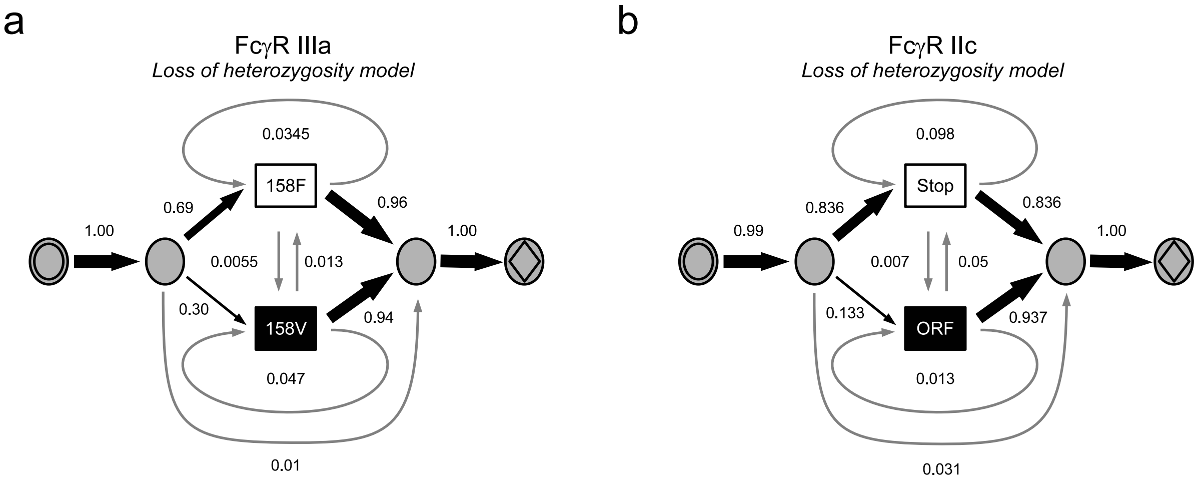

Suppl. Fig. 3 – **HMM models for FcγR IIIa and IIc to represent loss of heterozygosity.** For the corresponding basic models, compare Fig. 3c and d. **a**, HMM for FcγR IIIa including repetition loops that are dependent on the allele, reproducing the loss of heterozygosity signature predicted for the microhomology-mediated break-induced repair mechanism (compare Fig. 2d). The Bayes factor for this model was $\frac{1}{20.4}$ compared to the basic model shown in Fig. 3c. **b**, corresponding HMM for FcγR IIc. The Bayes factor for this model was $\frac{1}{11}$ compared to the basic model shown in Fig. 3d. The graphical representation is as described in Fig. 3.

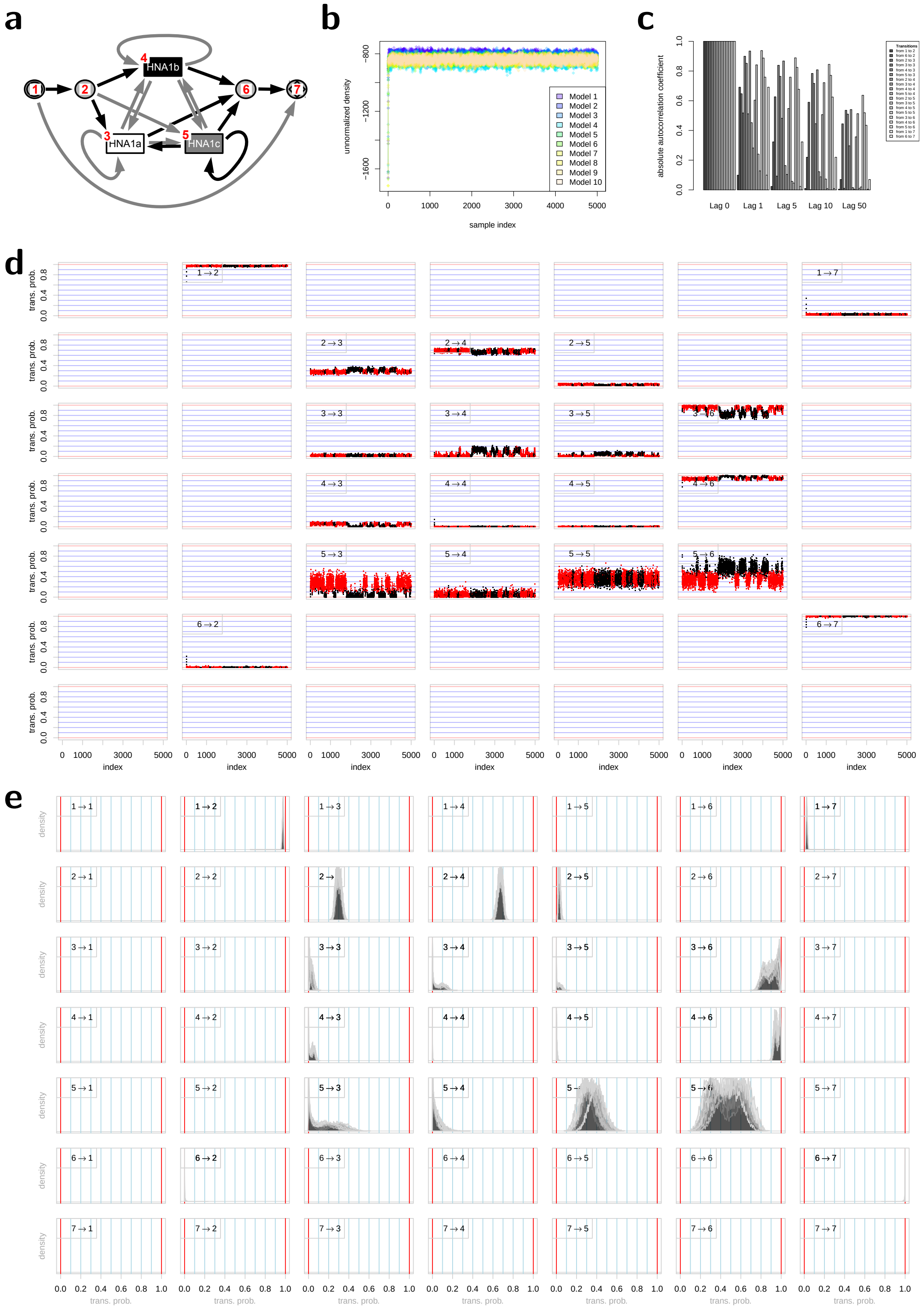

SUPPL. FIG. 4 – **Analysis of sampler output for the improved HMM for FcγR IIIb.** **a**, state numbering scheme of the HMM graph depicted in Fig. 4a and b. **b**, trace of non-normalized density values of a single sampler run. **c**, autocorrelation plot of a single sampler run for each varying transition probability. The bi-modal posterior parameter distribution causes an artifact in the calculation of autocorrelation times. **d**, trace plot of transition probabilities from a single sampler run. Each dot in the graph is colored according to a k-means cluster analysis (with k=2) to indicate the two modes of the posterior distribution. The plots are oriented according to the transition matrix, with each row indicating the first state and each column the second state of a one-step transition. **e**, corresponding density plots of transition probabilities using a Gaussian kernel estimator for each marginalized parameter.

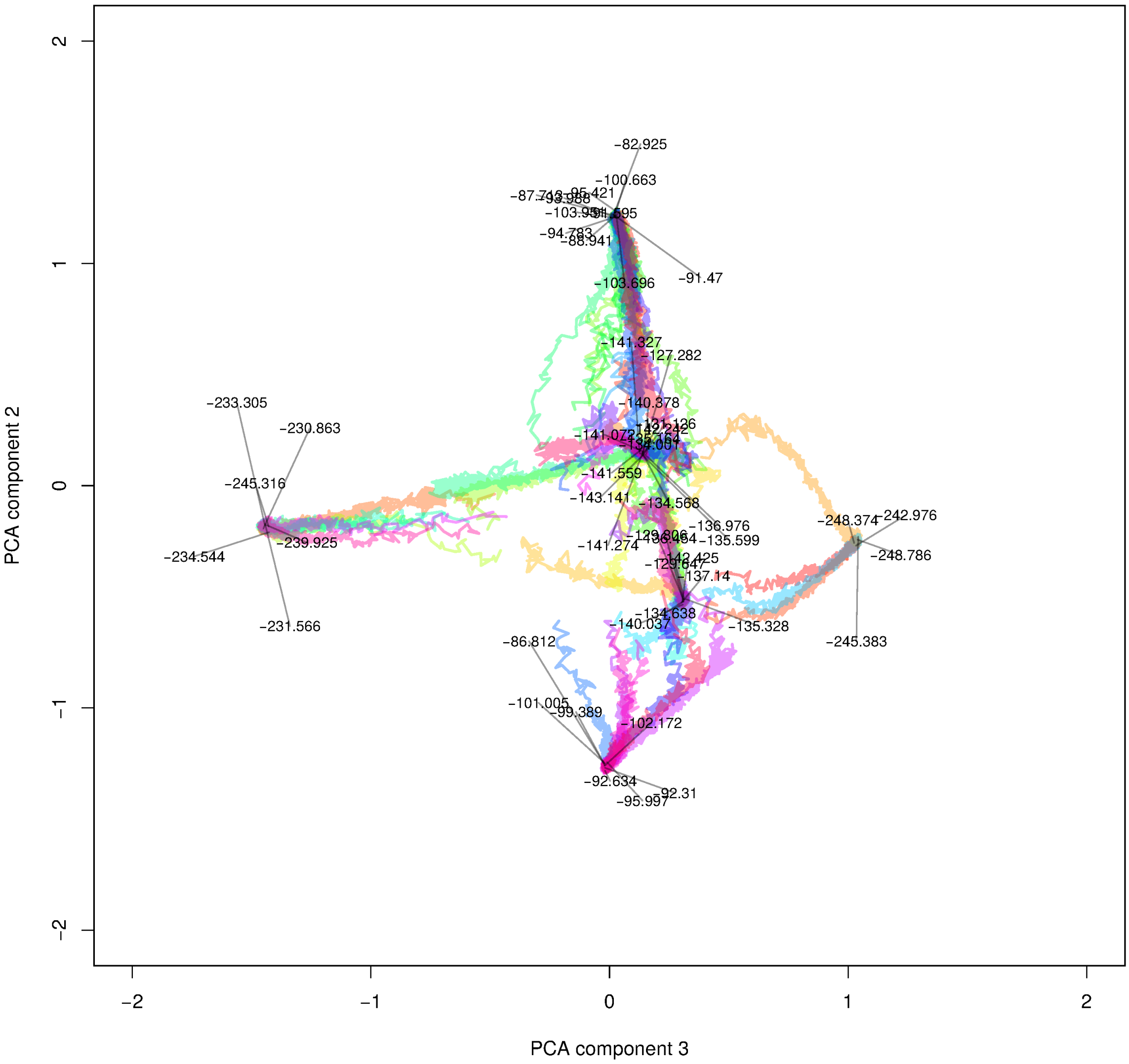

Suppl. Fig. 5 – **Visualization of MCMC traces for the linkage model of FcγR IIIa, IIc and IIIb copy numbers.** For the model shown in Fig. 6, the Metropolis-within-Gibbs sampling was repeated 50 times, and 3000 samples were recorded in each run. To map the parameter space in two dimensions and to visualize the 6 separate optima, the second and third components of a PCA of combined samples are shown. For each sampler run, a different color is chosen, with increasing saturation to indicate the ordering of samples. Chib and Jeliazkov marginal likelihoods for each trace are indicated at the convergence point of each trace.

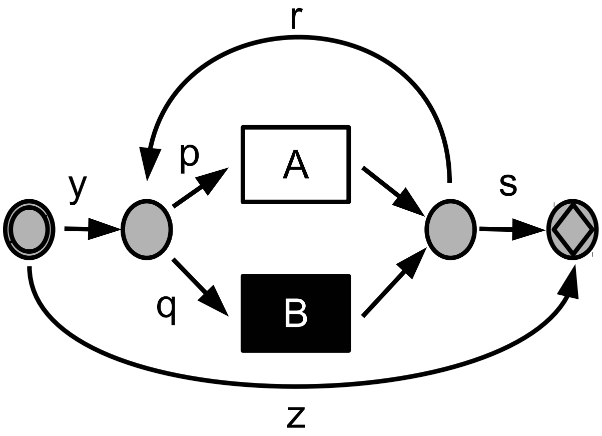

SUPPL. FIG. 6 – **Annotated HMM graph for Suppl. Tab. 1.** Corresponding graph in case of 2 alleles for the probability variables used in Suppl. Tab. 1.

Supplementary Table 1. Representation of genotype data as annotated flat table.

	Gene A		Gene B			Gene C	
	A_1	A_2	B_1	B_2	B_3	C_1	C_2
Individual 1	2	0	1	2	0	1	1
Individual 2	1	1	1	0	1	0	2
...	...	...	...	...	...	...	...

Supplementary Table 2. Regular Hardy-Weinberg equilibrium combinatorics

Genotype	Combinations	Probability[a]	Example[b]	Sum of probabilities	Example sums
`AA`	`A/A`	**p p**	0.64	$\mathbf{p}^2$	0.64
`AB`	`A/B`<br>`B/A`	**p q**<br>**q p**	0.16<br>0.16	**2pq**	0.32
`BB`	`B/B`	**q q**	0.04	$\mathbf{q}^2$	0.04

[a] p, probability of allele A; q, probability of allele B;
[b] Example probabilities `A` ($p = 0.8$), `B` ($q = 0.2$)

Supplementary Table 3. Extended Hardy-Weinberg equilibrium combinatorics

Genotype	Combinations	Probability[a]	Example[b]	Sum of probabilities	Example sums
0	0/0	$z\,z$	0.01	z^2	0.01
A	A/0	$y\mathbf{p}s\,z$	0.0576	$\mathbf{p}2ysz$	0.1152
	0/A	$z\,y\mathbf{p}s$	0.0576		
B	A/0	$y\mathbf{q}s\,z$	0.0144	$\mathbf{q}2ysz$	0.0288
	0/B	$z\,y\mathbf{q}s$	0.0144		
AA	A/A	$y\mathbf{p}s\,y\mathbf{p}s$	0.331776	$\mathbf{p}^2ys(ys+2zr)$	0.350208
	AA/0	$y\mathbf{p}r\mathbf{p}s\,z$	0.009216		
	0/AA	$z\,y\mathbf{p}r\mathbf{p}s$	0.009216		
AB	A/B	$y\mathbf{p}s\,y\mathbf{p}s$	0.082944	$2\mathbf{pq}ys(ys+2zr)$	0.175104
	B/A	$y\mathbf{q}s\,y\mathbf{p}s$	0.082944		
	AB/0	$y\mathbf{p}r\mathbf{q}s\,z$	0.002304		
	BA/0	$y\mathbf{q}r\mathbf{p}s\,z$	0.002304		
	0/AB	$z\,y\mathbf{p}r\mathbf{q}s$	0.002304		
	0/BA	$z\,y\mathbf{q}r\mathbf{p}s$	0.002304		
BB	B/B	$y\mathbf{q}s\,y\mathbf{q}s$	0.020736	$\mathbf{q}^2ys(ys+2zr)$	0.021888
	BB/0	$y\mathbf{q}r\mathbf{q}s\,z$	0.000576		
	0/BB	$z\,y\mathbf{q}r\mathbf{q}s$	0.000576		
AAA	AAA/0	$y\mathbf{p}r\mathbf{p}r\mathbf{p}s\,z$	0.00147456	$\mathbf{p}^3yrs(2ys+2zr)$	0.10911744
	AA/A	$y\mathbf{p}r\mathbf{p}s\,y\mathbf{p}s$	0.05308416		
	A/AA	$y\mathbf{p}s\,y\mathbf{p}r\mathbf{p}s$	0.05308416		
	0/AAA	$z\,y\mathbf{p}r\mathbf{p}r\mathbf{p}s$	0.00147456		
AAB	AAB/0	$y\mathbf{p}r\mathbf{p}r\mathbf{q}s\,z$	0.00036864	$3\mathbf{p}^2\mathbf{q}yrs(2ys+2zr)$	0.08183808
	ABA/0	$y\mathbf{p}r\mathbf{q}r\mathbf{p}s\,z$	0.00036864		
	BAA/0	$y\mathbf{q}r\mathbf{p}r\mathbf{p}s\,z$	0.00036864		
	AA/B	$y\mathbf{p}r\mathbf{p}s\,y\mathbf{q}s$	0.01327104		
	AB/A	$y\mathbf{p}r\mathbf{q}s\,y\mathbf{p}s$	0.01327104		
	BA/A	$y\mathbf{q}r\mathbf{p}s\,y\mathbf{p}s$	0.01327104		
	A/AB	$y\mathbf{p}s\,y\mathbf{p}r\mathbf{q}s$	0.01327104		
	A/BA	$y\mathbf{p}s\,y\mathbf{q}r\mathbf{p}s$	0.01327104		
	B/AA	$y\mathbf{q}sy\mathbf{p}r\mathbf{p}s$	0.01327104		
	0/AAB	$z\,y\mathbf{p}r\mathbf{p}r\mathbf{q}s$	0.00036864		
	0/ABA	$z\,y\mathbf{p}r\mathbf{q}r\mathbf{p}s$	0.00036864		
	0/BAA	$z\,y\mathbf{q}r\mathbf{p}r\mathbf{p}s$	0.00036864		
ABB	ABB/0	$y\mathbf{p}r\mathbf{q}r\mathbf{q}s\,z$	0.00009216	$3\mathbf{pq}^2yrs(2ys+2zr)$	0.02045952
	BBA/0	$y\mathbf{q}r\mathbf{q}r\mathbf{p}s\,z$	0.00009216		
	BAB/0	$y\mathbf{q}r\mathbf{p}r\mathbf{q}s\,z$	0.00009216		
	AB/B	$y\mathbf{p}r\mathbf{q}s\,y\mathbf{q}s$	0.00331776		
	BA/B	$y\mathbf{q}r\mathbf{p}s\,y\mathbf{q}s$	0.00331776		
	BB/A	$y\mathbf{q}r\mathbf{q}s\,y\mathbf{p}s$	0.00331776		
	A/BB	$y\mathbf{p}s\,y\mathbf{q}r\mathbf{q}s$	0.00331776		
	B/BA	$y\mathbf{q}s\,y\mathbf{q}r\mathbf{p}s$	0.00331776		
	B/AB	$y\mathbf{q}s\,y\mathbf{p}r\mathbf{q}s$	0.00331776		
	0/ABB	$z\,y\mathbf{p}r\mathbf{q}r\mathbf{q}s$	0.00009216		
	0/BBA	$z\,y\mathbf{q}r\mathbf{q}r\mathbf{p}s$	0.00009216		
	0/BAB	$z\,y\mathbf{q}r\mathbf{p}r\mathbf{q}s$	0.00009216		
BBB	BBB/0	$y\mathbf{q}r\mathbf{q}r\mathbf{q}s\,z$	0.00002304	$\mathbf{q}^3yrs(2ys+2zr)$	0.00170496
	BB/B	$y\mathbf{q}r\mathbf{q}s\,y\mathbf{q}s$	0.00082944		
	B/BB	$y\mathbf{q}s\,y\mathbf{q}r\mathbf{q}s$	0.00082944		
	0/BBB	$z\,y\mathbf{q}r\mathbf{q}r\mathbf{q}s$	0.00002304		
...	...	...	...	...	...

[a] Probabilities are equivalent to corresponding transition probabilities of the basic CNAV-reproducing HMM. p, probability of allele A; q, probability of allele B; r, probability of copy number gain; $s=1-r$; z, probability of deletion; $y=1-z$; compare Suppl. Fig. 6

[b] Example probabilities A ($p=0.8$), B ($q=0.2$), $z=0.1$, $r=0.2$

Supplementary Table 4. Description of mathematical symbols

Symbol	Description
Symbols for representation of genotypes	
$g_i \in \mathbb{N}_0$	component of vector $\mathbf{g}$, counting the abundance of allele i of a gene
$\mathbf{g} \in \mathbb{N}_0^l$	vector of length l of a single gene describing the abundance count of all gene alleles
$\mathbf{G} \in \mathcal{G}$	a k-tuple of vectors $\mathbf{g}$ to combine multiple genes into one genotype
$\mathbf{H} \in \mathcal{G}$	this symbol is used to indicate that a haplotype or single chromosome is described
$\mathcal{G}$	the set containing all technically possible values of $\mathbf{G}$
$\mathcal{O}$	the neutral element of $\mathcal{G}$
$\mathcal{F} = (\mathbf{G}_i)_{i=1,\dots,N}$	a family to represent a genotyping data collection
Symbols for representation of Markov models	
$\lambda = (S, V, A, B, \pi)$	Hidden Markov Model defined by a quintuple of parameters (S, V, A, B, π)
$S = \{s_1, \dots, s_n\}$	the set of hidden states of a hidden Markov models
$V = \{\mathbf{v}_1, \dots, \mathbf{v}_m\},\ \mathbf{v}_1, \dots, \mathbf{v}_m \in \mathcal{G}$	the alphabet set of possible emissions
$A \in \mathbb{R}^{n \times n}$	transition probability matrix, each row representing one state s_i, and each column the following states $s_1, \dots, s_n$
$B \in \mathbb{R}^{n \times m}$	emission probability matrix, each row representing one state s_i, and each column one element of V
$\mathbf{X} = (\mathbf{x}_t)_{1,\dots,T}$	a Markov path of length T, with states $\mathbf{x}_t \in S$
$\mathbf{Y} = (\mathbf{y}_t)_{1,\dots,T}$	corresponding emissions of a Markov path of length T, with $\mathbf{y}_i \in V$
$\mathcal{X}$	the set of admissible Markov paths
$\mathcal{X}^2 = \mathcal{X} \times \mathcal{X}$	the set of admissible Markov path *pairs*
${\mathcal{X}^2}_{\mathbf{G}} \subseteq \mathcal{X}^2$	the set of admissible Markov paths *pairs* that produce a certain genotype $\mathbf{G}$
Symbols for representation random variables and distributions	
$\Xi = [\xi_1, \dots, \xi_n]^T$	a squared matrix Ξ with n rows, consisting of row vectors ξ_i
$M(n, (p_1, \dots, p_k))$	the multinomial distribution with parameters n and probabilities $p_1, \dots, p_k$ for k categories
$Dirichlet(\alpha)$	the Dirichlet distribution with parameter vector α, used as conjugate prior for the multinomial distribution
$MatrixDirichlet(\Xi)$	the MatrixDirichlet distribution with an n-row parameter matrix Ξ, reflecting n independent Dirichlet distributions with parameter vector ξ_i

Symbol	Description
$B(\alpha)$	the multinomial Beta function, for vector parameter α
$B(\Xi) = \prod_{i=1}^{n} B(\xi_i)$	the extension of the multinomial Beta function with matrix parameter Ξ
$\mathbf{C}(\mathbf{X})$	the sufficient statistic of Markov path $\mathbf{X}$, summarizing the number of one-step transitions
$C = (c_{ij}) = \mathbf{C}(\mathbf{X}_1) + \mathbf{C}(\mathbf{X}_2) + \dots$	matrix representation of this summary statistic for multiple Markov paths
$\text{Prob}(\mathbf{X}\|A)$	the probability of a single Markov path $\mathbf{X}$, given the transition probabilities matrix A
$\text{Prob}(\mathbf{G}\|A)$	the probability of a genotype $\mathbf{G}$, given the transition probabilities matrix A
Symbols for the squirrel algorithm	
$(\mathbf{X}_1, \mathbf{X}_2) \in \mathcal{X}^2{}_{\mathbf{G}}$	a Markov path *pair* producing genotype $\mathbf{G}$, consisting of two independent Markov paths $\mathbf{X}_1$ and $\mathbf{X}_2$
$\mathrm{S}^{(\text{-}1)}((\mathbf{X}_1, \mathbf{X}_2)) \in \mathcal{X}^2{}_{\mathbf{G}}$	the direct predecessor of $(\mathbf{X}_1, \mathbf{X}_2)$, given a search tree ordered by a pseudo-random number generator
$\mathrm{S}^{(+1)}((\mathbf{X}_1, \mathbf{X}_2)) \in \mathcal{X}^2{}_{\mathbf{G}}$	the direct successor of $(\mathbf{X}_1, \mathbf{X}_2)$, given a search tree ordered by a pseudo-random number generator
$\mathrm{S}^{(\text{-n})}((\mathbf{X}_1, \mathbf{X}_2)) \in \mathcal{X}^2{}_{\mathbf{G}}$	n*th* predecessor of $(\mathbf{X}_1, \mathbf{X}_2)$
$\mathrm{S}^{(+\text{n})}((\mathbf{X}_1, \mathbf{X}_2)) \in \mathcal{X}^2{}_{\mathbf{G}}$	n*th* successor of $(\mathbf{X}_1, \mathbf{X}_2)$
